# Risk assessment of ‘*Candidatus* Liberibacter solanacearum’ transmission by the psyllids *Bactericera trigonica* and *B. tremblayi* from Apiaceae crops to potato

**DOI:** 10.1038/srep45534

**Published:** 2017-04-03

**Authors:** C. A. Antolinez, A. Fereres, A. Moreno

**Affiliations:** 1 Instituto de Ciencias Agrarias (ICA, CSIC), Consejo Superior de Investigaciones Científicas (CSIC), C/Serrano 115 Dpdo 28006 Madrid, Spain

## Abstract

*Candidatus* Liberibacter solanacearum (Lso) is bacterium transmitted by psyllids to Solanaceae and Apiaceae plants. So far, Lso is found in Europe affecting Apiaceae. In the Mediterranean region, *Bactericera trigonica* is the only known vector of Lso, but the leek-onion psyllid *Bactericera tremblayi* is another widespread psyllid and potential vector of Lso. Commonly, carrot, leek and potato are cultivated in the same zones and it is uncertain if these psyllid species are able to transmit Lso to potato plants. Here, we assessed the transmission of Lso by *B. trigonica* and *B. tremblayi* to potato plants. *B. trigonica* showed preference to ingest from the phloem, settle and oviposit on carrot and celery but not on potato. This was correlated with high Lso transmission rates to both carrot (80%) and celery (70%) but very low to potato (≤3%). *B. tremblayi* preferred leek over carrot and potato, the latter being the less preferred host. *B. tremblayi* readily ingested from the phloem of infected carrots but failed to transmit Lso from carrot to carrot. Our study shows that the risk of Lso transmission from Apiaceae to potato by *B. trigonica* is very low, and that *B. tremblayi* is not a likely vector of Lso.

Most plant pathogenic viruses and bacteria depend on insect vectors to spread to new areas or new hosts^1^. Consequently, vector feeding preferences are strongly correlated with the epidemiology of vector-transmitted plant pathogens^2^. The psyllids (Hemiptera, Psylloidea) are insect pests that ingest sap from the xylem and phloem tissues^3^,^4^,^5^. Nevertheless, they are commonly known as phloem feeders because nutrients obtained from this tissue are mandatory in order to complete their life cycle on certain host plants^6^. Actually, psyllid host range is restricted to only a few species or genera of plants^7^,^8^. Because of their feeding habits, psyllids could serve as vectors of phloem-restricted pathogens that cause economic losses in agriculture^8^.

In recent years, psyllids have been described transmitting the phloem-restricted bacterium *Candidatus* Liberibacter solanacearum (Lso), which is an emerging pathogen that causes severe losses primarily in potato and carrot^9^,^10^,^11^. Notably, different psyllid species transmit different haplotypes of Lso in different geographical areas and to different host plants^12^. In the Americas and New Zealand, Lso haplotypes A and B are transmitted to plants in Solanaceae by the potato psyllid *Bactericera cockerelli*^12^. In Europe and North Africa, Lso haplotypes C, D and E are transmitted to plants in Apiaceae by the carrot psyllids *Bactericera trigonica* and *Trioza apicalis*^13^,^14^. *Trioza apicalis* is found in northern and central European countries associated with Lso haplotype C, whereas *B. trigonica* is found in the Mediterranean region associated with Lso haplotypes D and E^10^,^14^. Despite the fact that it has been demonstrated that Lso could be transmitted through seeds, the main spread of this bacterium in the field is guided by psyllids that transmit the disease in a persistent-propagative manner^9^,^15^,^16^. Accordingly, Lso needs to replicate in the psyllid body and then inoculated into the plant with the saliva, when the psyllid feeds from the phloem sieve elements of a susceptible plant^5^,^17^,^18^. Therefore, success and efficiency of Lso transmission by psyllids heavily depends on vector feeding behaviour and vector host range.

The most powerful tool to study feeding behaviour of hemipterans is the electrical penetration graph (EPG) technique^19^,^20^,^21^. This technique has been used to correlate psyllid-feeding activities with the transmission of different species of *Candidatus* Liberibacter^3^,^5^,^18^,^22^,^23^. Recently, Munyaneza *et al*.^22^ used EPG to assess the risk of cross transmission of Lso from potato to carrot by the potato psyllid *B. cockerelli*^22^. In that study, *B. cockerelli* had difficulty reaching the phloem tissues of carrot plants, which was correlated with very low percentages of Lso transmission in carrots. Additionally, Munyaneza *et al*.^22^ also showed that Lso haplotype B from potatoes produces symptoms in carrots. Therefore, despite the fact that Lso haplotypes associated with potato can infect carrots, the movement of Lso from potato to carrot plants mediated by *B. cockerelli* is unlikely because carrot is not a host of this psyllid species.

While the risk of transmission from potato to carrot mediated by *B. cockerelli* has been assessed, the risk of Lso transmission from carrot to potato or to other crops by carrot psyllids in Europe is unknown. In the Mediterranean region for example, the carrot psyllid *B. trigonica* is the only known vector of Lso^14^,^24^. Nevertheless, it is unknown if *B. trigonica* is able to feed on potatoes and subsequently can transmit Lso to this crop. In addition to *B. trigonica*, other psyllid species (*Bactericera tremblayi* and *Bactericera nigricornis*) are common in the Mediterranean region and have been suggested as potential vectors of Lso in Spain^24^. So far, *B. nigricornis* and *B. tremblayi* have been found in very low populations in carrot and potato fields^24^. However *B. tremblayi* has been reported as a serious pest in onion and leek crops^25^, suggesting this psyllid may constitute a potential treat to carrot or potato because leek and onion fields are commonly grown in the vicinity of carrot and potato crops.

Despite of its economic importance, the potential risk of cross transmission of Lso from Apiaceae to potato by *B. trigonica* has not been evaluated. Moreover, the role of *B. tremblayi* as an Lso vector remains uncertain and must be determined. Thus, the main objective of this study was to evaluate the risk of Lso cross transmission from Apiaceae to potato and other crops mediated by *B. trigonica* and *B. tremblayi* two of the most abundant psyllid species in Spain. Our specific objectives were: i) to evaluate the potential of Lso transmission to potato, carrot, celery and leek by assessing the probing behaviour of *B. trigonica* and *B. tremblayi* using the EPG technique, ii) to assess whether *B. tremblayi* is a vector of Lso, and iii) to assess the settling preference and Lso transmission of *B. trigonica* (in carrot celery and potato) and *B. tremblayi* (in potato, carrot and leek) by performing non-choice and dual-choice assays.

## Results

### Probing behaviour of *Bactericera trigonica* and *Bactericera tremblayi* on different hosts

Stylet penetration process of *B. trigonica* and *B. tremblayi* in different host plants was monitored using the electrical penetration graph technique (EPG). The probing duration per insect (PDI) showed no differences between carrot and celery but was shorter when *B. trigonica* probed on potato than on carrot or celery, (F = 7.17, df = 2, 47, P = 0.002) (Table 1). In contrast to the PDI, the time from the first probe to the first phloem salivation (E1) was longer for insects probing on potato than for those on carrot or celery (F = 8.10, df = 2, 47 P = 0.001) (Table 1). *Bactericera trigonica* spent more time in non-probing activities (NP) on potato compared with celery or carrot (F = 5.71, df = 2, 47, P = 0.006). The duration of the stylet pathway (C) and the time to the first probe from the start of the EPG were not significantly different when *B. trigonica* probed on carrot, potato or celery (Duration of C: F = 0.97, df = 2, 47, P = 0.385; Time to the first probe from the start of EPG: H = 2.88, df = 2, P = 0.236) (Table 1). On the other hand, *B. trigonica* probed in similar proportions from the xylem tissues (G waveform) of celery and potato but probed more from xylem of carrot compared to potato. (Number of G: H = 12.09, df = 2, P = 0.002). Additionally, *B. trigonica* failed to reach the phloem tissues of potato plants because no phloem salivation (E1) or ingestion (E2) waveforms were detected. By contrast, E1 and E2 waveforms were commonly detected when *B. trigonica* probed carrot or celery (Table 1).

For *B. tremblayi*, the time to the first probe from the start of the EPG was different between leek and potato but not between leek and carrot or when comparing carrot and potato (H = 10.38, df = 2, P = 0.006) (Table 1). The duration of stylet pathway (C) was similar in all plants tested (F = 2.00, df = 2, 47, P = 0.140). The duration of non-probing (NP) and the time from the first probe to the first phloem salivation (E1) were significantly shorter in leek than those in carrot or potato and no differences were observed between carrot and potato (Duration of NP: H = 23.85, df = 2, P = 0.000; Time from first probe to first E1: H = 17.87, df = 2, P = 0.000) (Table 1). Probing duration per insect (PDI) was higher in leek than that in carrot or potato and no differences were detected between carrot and potato (F = 9.71, df = 2, 47, P = 0.000) (Table 1). Data showed that EPG variables related to xylem activities were similar among all species of plants tested (Number of G: H = 1.81, df = 2, P = 0.403; Duration of G: F = 0.30, df = 2, 47 P = 0.739) (Table 1). Moreover, *B. tremblayi* reached the phloem tissues of all plants tested but showed a clear preference to probe longer and to spend more time in phloem-associated waveforms on leek than on carrot or potato (Number of E1: H = 21.27, df = 2, P = 0.000; Duration of E1: H = 28.94, df = 2, P = 0.000; Duration of E2: H = 30.50, df = 2, P = 0.000) (Table 1). Additionally, although not statistically significant, *B. tremblayi* reached the phloem salivation phase (E1) more frequently on carrot than on potato (Table 1). Furthermore, *B. tremblayi* ingested phloem sap (E2) from carrots but failed to ingest phloem sap from potatoes (Table 1).

### Transmission of Lso by *Bactericera tremblayi* from carrot to carrot

Lso transmission efficacy by *B. tremblayi* from carrot to carrot was assessed in greenhouse tests. Only 11% of the *B. tremblayi* individuals acquired Lso (44 Lso positive insects/400 insects analysed) after 72 h exposure to Lso-infected carrots. Notably, after a 15 d post-acquisition period, none of the psyllids that acquired Lso inoculated healthy carrot plants with the bacteria (0 infected plants/30 receptor plants with at least one infected psyllid per plant). Therefore, although the results showed clearly that *B. tremblayi* acquired the bacterium, the pathogen was not transmitted to carrots by this species of psyllid.

### Settling preference, oviposition and Lso transmission on different hosts

#### Bactericera trigonica

**Figure 1:**
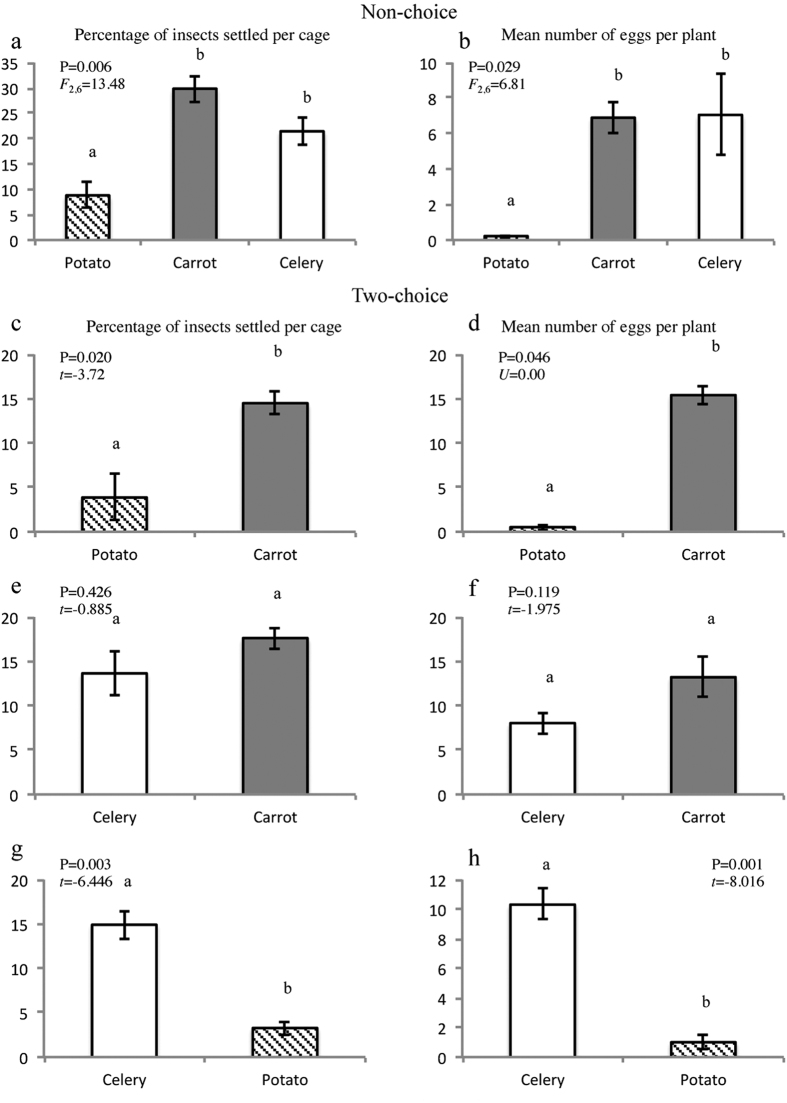
Settling and oviposition preferences of *B. trigonica* on potato, carrot and celery in non-choice assays: (**a**) Percentage of insects settled and (**b**) Number of eggs per plant. Different letters represent significant differences among treatments according to one-way ANOVA. Settling and oviposition preferences of *B. trigonica* in dual-choice assays: (**c**) Percentage of insects settled on celery and carrot; (**d**) Number of eggs per plant on celery and carrot; (**e**) Percentage of insects settled on celery and potato; and (**f**) Number of eggs per plant on celery and potato. Different letters represent significant differences according to Student’s *t*-tests or Mann-Whitney U-tests. Error bars represent standard error.

Settling preferences and oviposition on carrot, celery and potato as well as Lso transmission by *B. trigonica* were evaluated in non-choice and dual-choice assays. Adult *B. trigonica* did not show a preference between the *Apiaceae* plants tested in the non-choice and dual-choice assays. In both assays, the settling and oviposition preference of *B. trigonica* was similar for carrot and celery (Fig. 1a,b,e,f). However, settling and oviposition were significantly reduced on potato compared with carrot or celery in both the non-choice and dual-choice assays (Fig. 1a,b,c,d,g,h). The percentage of infested plants was also higher for carrot or celery than that for potato, but when comparing carrot and celery in both non-choice and dual-choice assays, no differences in the percentage of infested plants were observed (Supplementary Table S1).

**Figure 2:**
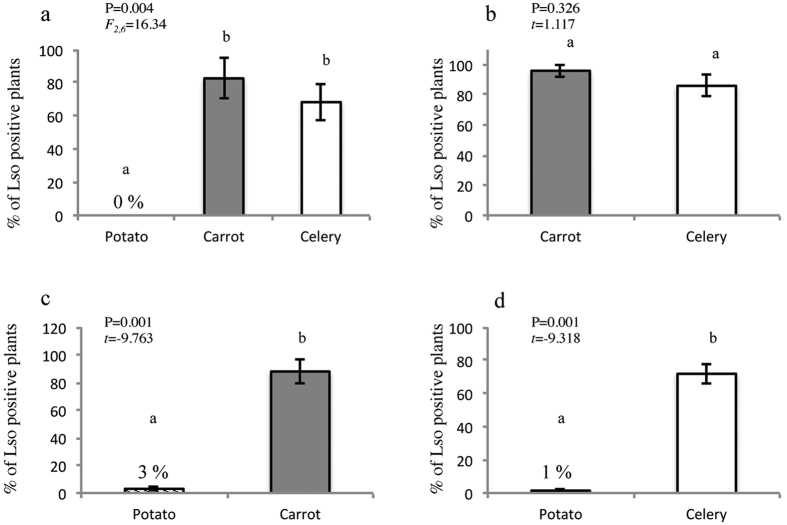
(**a**) Percentage of infected plants when *B. trigonica* transmitted Lso in no-choice assays. Different letters represent significant differences according to one-way ANOVA. Percentage of infected plants when *B. trigonica* transmitted Lso in dual-choice assays: (**b**) Carrot-celery, (**c**) Potato-carrot, and (**d**) Potato-celery. Different letters represent significant differences according to Student´s *t*-tests.

The transmission of Lso to the different plants tested in both non-choice and dual-choice assays was different for *B. trigonica* (Fig. 2). In non-choice tests, *B. trigonica* did not transmit Lso to potatoes (Fig. 2a). By contrast, *B. trigonica* efficiently transmitted Lso to carrot and celery, which showed similar percentages of transmission (Fig. 2a). In potato-carrot and potato-celery treatments in the dual-choice assay, Lso transmission was always significantly higher to carrot or celery than to potato (Fig. 2c,d). However, in the carrot-celery treatment, Lso transmission was not significantly different between the two plant hosts (Fig. 2b).

#### Bactericera tremblayi

**Figure 3:**
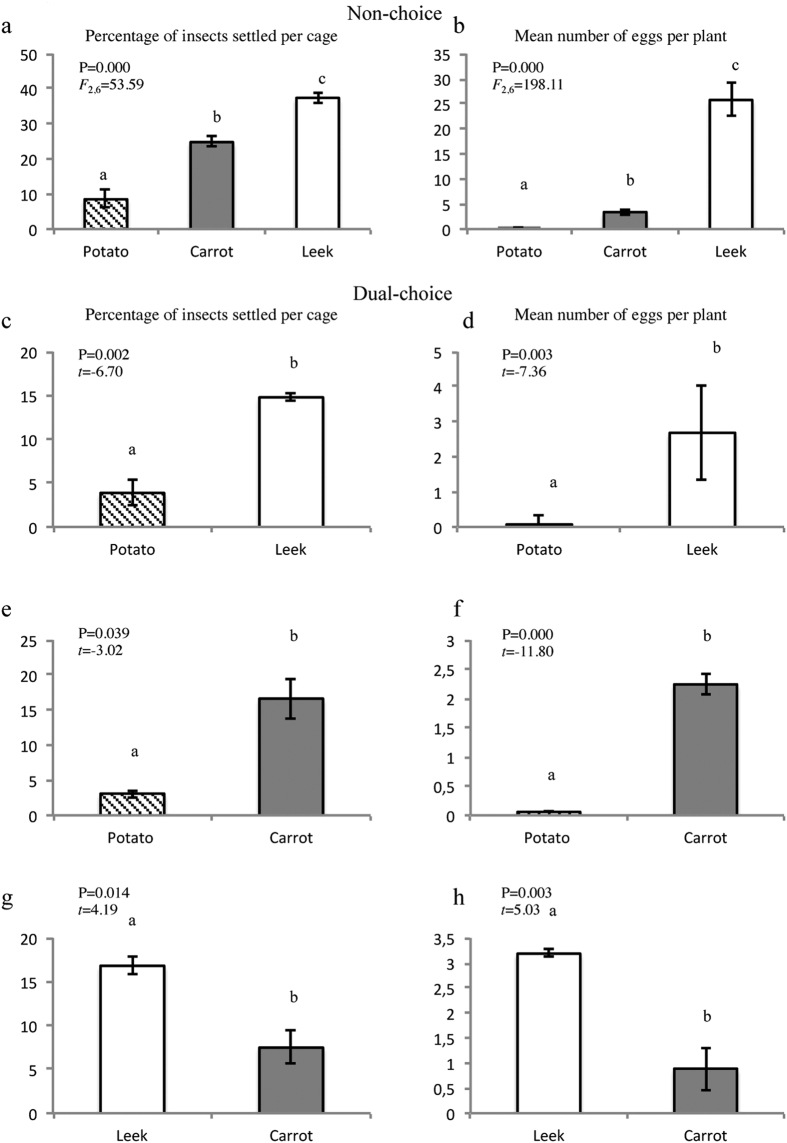
Settling and oviposition preferences of *B. tremblayi* on leek, carrot or potato in non-choice assays. (**a**) Percentage of insects settled and (**b**) Number of eggs per plant. Different letters represent significant differences among treatments according to one-way ANOVA. Settling and oviposition preferences of *B. trigonica* in dual-choice assays: (**c**) Percentage of insects settled on potato and leek; (**d**) Number of eggs per plant on potato and leek; (**e**) Percentage of insects settled on potato and carrot; (**f**) Number of eggs per plant on potato and carrot; (**g**) Percentage of insects settled on leek and carrot; and (**h**) Number of eggs per plant on leek and carrot. Different letters represent significant differences according to Student’s *t*-tests. Error bars represent standard error.

Settling and oviposition of *B. tremblayi* on leek, carrot and potato were also evaluated in non-choice and dual-choice assays. Settling preference of *B. tremblayi* was significantly different among all host plants tested in the non-choice and dual-choice assays (Fig. 3). Our data indicated that *B. tremblayi* preferred to settle and oviposit on leek compared with carrot or potato in both types of assay (Fig. 3a,b,c,d,g,h). Additionally, *B. tremblayi* preferred to settle and oviposit on carrot compared with potato (Fig. 3a,b,e,f). Compared with potato, a higher percentage of leek or carrot were infested, although no differences were detected between carrot and leek (Supplementary Table S2).

## Discussion

Lso is a persistently transmitted pathogen that has a close relationship with the psyllid vectors^15^,^17^. For successful transmission, the stylet of Lso infected psyllids must reach and inoculate infected saliva into the phloem tissues of a susceptible host plant^5^,^18^,^22^. Because of this, the study of psyllid host preferences and stylet penetration process is fundamental for a complete understanding of the epidemiology of Lso in different crops. In this work, we showed that settling, oviposition and feeding behaviour patterns were related to the propensity of the vectors *B. trigonica* and *B. tremblayi* to transmit *Lso* to Apiaceae or potato plants, respectively.

In this study, *B. trigonica* clearly preferred to settle and oviposit on Apiaceae compared with potato plants. Settling and oviposition of *B. trigonica* on potato was very low, even when other plant species were not available (non-choice assay). The EPG data were consistent with this marked preference pattern for Apiaceae by *B. trigonica* and demonstrated that *B. trigonica* engaged in sustained feeding from the phloem of carrot and celery but failed to reach and feed from the phloem of potato plants. Since psyllids use the phloem sap as the primary source of sugars and amino-acids^6^, feeding from phloem tissues is fundamental for the reproduction and the ability of a psyllid species in order to colonize a given host. Therefore, this information suggests that *B. trigonica* cannot colonize potato because it could not continuously feed from the phloem tissues of potato plants.

On the other hand, phloem feeding is an absolute prerequisite for transmission, thus only colonizing vectors capable of sustained phloem feeding can efficiently spread phloem-restricted pathogens^26^,^27^. Actually, inoculation success is directly correlated with the duration of the salivation phase performed by the vector just before phloem ingestion^5^,^28^. Our results support these assumptions because high Lso transmission rates were detected in plants on which *B. trigonica* settled for long periods and salivated and ingested from the phloem. However, null or very low Lso transmission was obtained for potato on which *B. trigonica* showed poor settling, low oviposition and neither salivation nor phloem ingestion. Interestingly, despite no phloem related activities were observed by EPGs in an 8 h period, a very low percentage of potato plants tested positive for Lso in the dual-choice assays. This result suggest that eventually a few individuals of *B. trigonica* may have reached the phloem of potato in the extended 72 h period used in our dual-choice assays.

According to the results of this study, the risk of Lso transmission mediated by *B. trigonica* from Apiaceae to potato would be very low. Although highly infected carrot and celery crops commonly overlap in growing zones with potato, our data suggest that *B. trigonica* may land on but would not settle or feed from the phloem of potato. This result is consistent with few *B. trigonica* captured in potato crops growing near to carrot crops^24^. With these conditions, the primary transmission of Lso mediated by *B. trigonica* would be very unlikely. Moreover, if primary transmission does occur, secondary dispersion from the infected potato to other potatoes is likely be very low because *B. trigonica* cannot feed in a sustained way from the phloem tissues and therefore cannot colonize potato.

Generally, the results of this work were consistent with and complements well the work performed by Munyaneza *et al*.^22^. They showed that the risk of Lso transmission from potato to carrot is negligible because the potato psyllid *B. cockerelli* is not able to efficiently localize and feed from the phloem tissues of carrot plants. In our study, the risk of movement of Lso mediated by *B. trigonica* from carrots to potato was very low for a similar reason. However, assessing the risk of cross transmission from carrot to potato in Europe is complex because more than one vector could be involved in the transmission of this pathogen. For example, the role of *T. apicalis* in cross transmission from carrot to other economically important crops in northern Europe remains unknown and requires further investigation.

Additionally, other psyllid species catalogued as potential vectors of Lso should receive special attention, e.g., *B. tremblayi* or *B. nigricornis*^24^. Our data showed that *B. tremblayi*, preferred to settle, oviposit and feed on leek but also settled and fed from phloem on carrots. This result was unexpected because the attempts to rear *B. tremblayi* on carrots were unsuccessful, and to our knowledge, the life cycle of this psyllid cannot be completed on carrot^7^. However, for psyllids, adults and nymphs may display different host plant specificity, and adults of some psyllid species may feed temporarily from plant species unsuitable for nymphal development^8^. Based on this scenario, our results suggest that in the absence of a plant suitable for reproduction, adults of *B. tremblayi* might use carrot as a temporary food plant. The EPG results support such use because *B. tremblayi* also ingested phloem sap from carrots. Although feeding from phloem was correlated with the capacity of this species to acquire Lso, we also showed that this psyllid species did not transmit the bacterium to carrot. Lack of transmission could be explained by different factors. For example, it has been reported that when Lso is acquired, the bacteria must pass through the insect midgut epithelium to infect the haemolymph and then enter the salivary glands^15^,^29^. It is uncertain if Lso is able to complete circulation in the body of *B. tremblayi* since this process has not been studied in this psyllid species. Finally, in this Lso-psyllid interaction, it is possible that longer latency periods than normal are required for the replication of Lso in the psyllid body, which provides another explanation for the absence of transmission. However, we did not test for latency periods in *B. tremblayi*, and the only reported latency period for Lso is in *B. cockerelli*^29^; therefore, additional research is recommended to fully understand the Lso infection process in this psyllid species.

In conclusion, our results suggest that the host plant preferences of *B. trigonica* strongly influences the host range of Lso in the Mediterranean region. As a consequence, the risk of Lso transmission from carrot to potato mediated by *B. trigonica* is negligible, but tests of the transmission ability of other psyllid species that feed on potato might identify other risks. Additionally, we concluded that the hypothesis was not supported that *B. tremblayi* is a competent vector of Lso.

## Methods

### Plants and insects

The following plant species were used in the assays: carrot (*Daucus carota,* variety ‘Bangor’), celery (*Apium graveolens,* variety ‘Pascal’), potato (*Solanum tuberosum*, variety ‘Monalisa’) and leek (*Allium porrum*, variety ‘Costeau’). Seeds of carrot (Bejo Zanden b. v. The Netherlands), leek (Royal Sluis SVS, Holland B. V), and celery (Battle S.A, Barcelona, Spain) and potato tubers were germinated individually in 8 cm diameter pots with soil substrate (Kekkilä Iberica, Almería, Spain). Plants were grown in an insect-proof chamber at 24:18 °C (L:D), 60–80% relative humidity (RH) and with a 16:8 h (L:D) photoperiod. Plants were watered three times a week with 20–20–20 (N:P:K) Nutrichem fertilizer (Miller Chemical & Fertilizer Corp., PA, USA) at a dose of 0.25 g L^−1^. Plants were maintained in the growth chamber until reaching the developmental stage of three true leaves for carrot, celery and leek and four leaves for potatoes.

Colonies of *B. trigonica* and *B. tremblayi* were maintained in the greenhouse facilities at ICA-CSIC, Madrid, Spain. The *B. trigonica* colony was collected from carrot fields in Gomezserracín, Segovia, Spain, in 2014. Individuals for the *B. tremblayi* colonies were also collected in Gomezserracín from commercial leek fields in 2014. Colonies of *B. trigonica* were reared on carrot plants in 47.5 × 47.5 × 47.5 cm cages (length × width × height; nylon mesh, 150 μm), and colonies of *B. tremblayi* were reared on leek plants in similar insect cages. Attempts to rear *B. trigonica* on plants other than *Apiaceae* or *B. tremblayi* on plants other than *Allium* were unsuccessful. Colonies of *B. trigonica* and *B. tremblayi* were housed in different glasshouses under similar greenhouse conditions, i.e., 26:18 °C (L:D) and a photoperiod of 16:8 h (L:D). Colonies were tested for Lso by real-time PCR; the percentage of infection of the *B. trigonica* colony was 97%, whereas *B. tremblayi* was Lso-free. Dr. Jaime Cubero from the National Institute for Agronomic Research (INIA), Madrid, Spain, kindly identified the Lso haplotype in our *B. trigonica* colonies as haplotype E, according to the procedure described by Nelson *et al*.^12^. For the assays, 5–7-d-old, adult psyllids were collected from the psyllid colonies with a handmade vacuum aspirator one hour after the beginning of the experiments. Groups of insects used in the preference and transmission assays contained similar proportions of males and females.

### EPG analysis of psyllid probing behaviour

The probing behaviour of *B. trigonica* and *B. tremblayi* was monitored on different plants (carrot, celery and potato for *B. trigonica* and carrot, leek and potato for *B. tremblayi*) using the electrical penetration graph (EPG) technique. A gold wire electrode (2 cm length, 20 μm diameter) was attached to the insect pronotum following the procedure described by Antolínez *et al*.^23^. A second electrode (copper, 10 cm length, 2 mm diameter) was inserted into the soil of the plant container. The psyllids were starved for 1 h during acclimatization between the time of wiring and the beginning of EPG recording. Then, the psyllids were placed on the abaxial surface of a fully expanded leaf and were allowed to probe and feed on the test plant for 8 h. The EPG recordings were obtained using an eight channel DC EPG system (type Giga-8; Wageningen University, The Netherlands). The signal was digitized using a DI-710 board (Dataq^®^ Instruments, Akron, OH, USA), and the digitized data were loaded onto a PC and analysed with Stylet+ software (EPG Systems, Wageningen, The Netherlands). A minimum number of 15 replicates were obtained for each plant and psyllid combination. EPG waveforms previously described for psyllids were identified according to Pearson *et al*.^4^ as follow: non-probing (NP), intercellular stylet pathway (C), salivation into phloem sieve elements (E1), phloem sap uptake from the sieve elements (E2) and active intake of xylem sap from xylem vessels (G). The following EPG parameters were calculated to describe pathway activity and phloem or xylem activity according to Backus *et al*.^30^: probing duration per insect, PDI, is the amount of time an average insect has the stylet inserted; number of waveform events per insect, NWEI, is the sum of the number of events of a particular waveform divided by the total number of insects in each treatment; total waveform duration (min) per insect, WDI, is the sum of durations of each event of a particular waveform divided by the total number of insects in each treatment; and waveform duration per event by insect, WDEi, is the average duration of events of a given waveform by an insect in a cohort. The variable time from the first probe to the first E1 was calculated according to Sarria *et al*.^31^.

### Lso detection by real-time PCR

To detect Lso in plants, plant DNA was purified following the CTAB (cetyltrimethyl ammonium bromide) protocol^32^. To detect Lso in psyllids, psyllid DNA was obtained following the squash protocol^33^. Then, Lso was detected by real-time PCR in plant and psyllids using the primers, TaqMan probe and procedure described by Bertolini *et al*.^16^.

### Transmission of Lso by *Bactericera tremblayi*

Adult psyllids of *B. tremblayi* were tested for both the acquisition of Lso from carrots and the inoculation of carrots with Lso. Groups of psyllids were exposed to Lso-infected carrot plants for an acquisition access period (AAP) of 72 h. Then, the psyllids were removed from carrots and transferred to leek plants for 15 d (latency period of Lso). After the 15 d, 400 psyllids were collected from the leek plants and transferred to 100 healthy carrot plants, each contained in a transparent, plastic cylindrical cage (4 psyllids/plant). The psyllids had access to the entire plant for an inoculation access period (IAP) of 24 h. Later, the psyllids were removed and tested individually using real-time PCR. Plants exposed to groups of insects that tested negative for Lso by real-time PCR (70 plants) were discarded from the analysis. Plants exposed to groups of insects that tested positive for Lso (30 plants) were sprayed with 1 g L^−1^ of Confidor ^®^ (Bayer, Kansas City, MO, USA) on days zero and 10 and were maintained under greenhouse conditions for eight weeks to test for Lso by visual inspection of symptoms and by real-time PCR.

### Settling preference, oviposition and Lso transmission on different hosts

#### Non-choice assays

The assays used a set of three cages with each cage containing one treatment. The following three treatments were evaluated: (T1) 36 carrot plants, (T2) 36 celery plants and (T3) 36 potato plants. Each treatment was replicated three times, and the replicates were rearranged to minimize location effects. Each cage contained potted plants arranged in a square (six rows and six columns, with 12.5 cm between plants). The cages were 1 × 1 × 1 m and covered with an aphid-proof mesh net. On a flight platform similar to that described by Fereres *et al*.^34^, two hundred Lso-infected individuals of *B. trigonica* were released. The flight platform was place 0.5 m above the test plants inside each cage. Insects were released at solar noon in greenhouse conditions similar to those described for insect rearing. All cages were rotated 180° daily to avoid orientation bias. The percentage of psyllids settled per test plant was determined, and the eggs per plant were counted 72 h after psyllid release. Settling preference was calculated as the percentage of insects settled on plants per cage. The percentage of plants infested by at least one insect was also calculated. Then, test plants were sprayed with 1 g L^−1^ of Confidor ^®^ (Bayer, Kansas City, MO, USA) and re-sprayed 10 d later to avoid further Lso transmission. Plants were maintained in a separate glasshouse under greenhouse conditions. Eight weeks after the experiment was completed, the percentage of plants infected with Lso was evaluated by visual inspection of symptoms and by real-time PCR as described above.

In a separate assay, the percentage of infested plants, settling and oviposition of *B. tremblayi* were also evaluated using a similar procedure to that described for *B. trigonica*. In these assays, the following treatments were evaluated: (T1) 36 leek plants, (T2) 36 carrot plants, and (T3) 36 celery plants. Because of the lack of transmission shown by *B. tremblayi* in the previous transmission experiments (see results section), the individuals of *B. tremblayi* used in this assay and in the dual-choice assay were Lso-free. Thus, Lso transmission was not evaluated for *B. tremblayi*. This assay was also repeated three times, and the parameters evaluated were identical to those described for *B. trigonica*.

#### Dual-choice assays

For these assays, we used a similar procedure to that described for the non-choice assays. However, each experimental arena contained a combination of two different plant species (18 plants of each species were alternately arranged in a square 6 × 6 layout, with 12.5 cm between plants) (Supplementary Fig. S1). To assess the preference of *B. trigonica*, the assay included three treatments of the following plant combinations, with each in a separate cage: (T1) carrot-celery, (T2) celery-potato and (T3) potato-carrot. Additionally, a separate assay was performed to assess the host plant preference of *B. tremblayi*. This assay included the following treatments: (T1) carrot-leek, (T2) carrot-potato, and (T3) leek-potato. The parameters that were evaluated and the methodology used in this assay were identical to those described for the non-choice assays.

#### Statistical analyses

All behavioural variables were tested for normality using the Shapiro-Wilk W-test and were transformed when required by either sqrt(x + 1) or ln(x + 1). Comparisons among EPG treatments were performed using one-way ANOVA for Gaussian variables or with Kruskal-Wallis tests when normality was not achieved. For the preference assays, the percentage of insects settled per cage, the percentage of plants infected with Lso and the percentage of infested plants were transformed by arcsin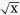 when required to reduce heteroscedasticity and achieve normality. Following transformation, values were rechecked for normality using Shapiro-Wilk W-tests, and then the means for each treatment were compared using one-way ANOVA for the non-choice tests. For the dual-choice tests, pairwise comparisons between combinations of plants in each treatment were performed with a Student’s t-test or with a Mann-Whitney U-test when normality was not achieved. Data were analysed using the SPSS 21 statistical software package (IBM Corp).

## Additional Information

**How to cite this article:** Antolinez, C. A. *et al*. Risk assessment of ‘*Candidatus* Liberibacter solanacearum’ transmission by the psyllids *Bactericera trigonica* and *B. tremblayi* from Apiaceae crops to potato. *Sci. Rep.* **7**, 45534; doi: 10.1038/srep45534 (2017).

**Publisher's note:** Springer Nature remains neutral with regard to jurisdictional claims in published maps and institutional affiliations.

## Supplementary Material

Supplementary Information

## Figures and Tables

**Table 1:** Means and SEs of probing behaviour variables for *Bactericera trigonica* on carrot, celery and potato and for *Bactericera tremblayi* on leek, carrot and potato.

	*B. trigonica*					*B. tremblayi*			
	EPG Parameter	Celery (n = 17)	Carrot (n = 18)	Potato (n = 15)	P value	Leek (n = 18)	Carrot (n = 17)	Potato (n = 15)	P value
Activities prior to vascular tissues	Time to 1^st^ probe from start of EPG (WDEi)	16,42 ± 6.74 a	12.30 ± 3.70 a	26.72 ± 5.99 a	0.236	5.85 ± 1.26 a	21.63 ± 9.84 ab	42.02 ± 9.26 b	0.006
	Duration of C (WDI)	163.02 ± 19.25 a	201.05 ± 21.73 a	202.25 ± 27.42 a	0.385	163.10 ± 27.14 a	238.44 ± 34.11 a	226,83 ± 24.74 a	0.140
	Duration of NP (WDI)	180.75 ± 20.25 a	140.57 ± 16.07 a	248.58 ± 27.42 b	0.006	54.60 ± 20.12 a	214.60 ± 25.28 b	226.07 ± 24.71 b	0.000
	Probing duration per insect (PDI)	299.21 ± 20.25 a	335.97 ± 18.89 a	219.44 ± 25.87 b	0.002	425.39 ± 20.12 a	293.62 ± 38.73 b	253.91 ± 24.71 b	0.000
	Time from 1^st^ probe to 1^st^ E1	249.41 ± 44.61 a	300.86 ± 35.39 a	445.23 ± 8.47 b	0.001	177.26 ± 35.09 a	397.97 ± 37.11 b	435.62 ± 8.82 b	0.000
Xylem activity	Number of G (NWEI)	1.17 ± 0.28 ab	1.50 ± 0.13 b	0.53 ± 0.16 a	0.002	1.00 ± 0.05 a	1.23 ± 0.16 a	1.00 ± 0.16 a	0.403
	Duration of G (WDI)	44.19 ± 12.45 ab	50.00 ± 11.11 b	17.18 ± 5.14 c	0.040	26.69 ± 3.19 a	34.99 ± 4.59 a	33.85 ± 4.93 a	0.739
Phloem Activity	Number of E1 (NWEI)	2.47 ± 0.76 a	1.88 ± 0.44 a	0 c	0.000	2.55 ± 0.50 a	1.35 ± 0.68 b	0.06 ± 0.06 b	0.000
	Duration of E1 (WDI)	25.36 ± 9.60 a	6.11 ± 2.43 a	0 c	0.000	15.52 ± 3.56 a	0.66 ± 0.38 b	0.07 ± 0.01 b	0.000
	Number of E2 (NWEI)	2.05 ± 0.66 a	1.61 ± 0.39 a	0 c	0.000	2.1 ± 0.36 a	1.35 ± 0.68 b	0 c	0.000
	Duration of E2 (WDI)	66.62 ± 21.15 a	78.80 ± 20.61 a	0 c	0.008	220.06 ± 18.33 a	25.59 ± 34.58 b	0 c	0.000

Different letters indicate significant differences among columns according to ANOVA or Kruskal-Wallis tests. Waveform definitions: **C**, intercellular stylet pathway; **G**, xylem ingestion; **E1**, salivation into phloem sieve elements; **E2** ingestion from sieve elements; and **NP**, non-probing. Number of waveforms performed by insect (NWEI); Waveform duration performed by insect (WDI); Probing duration per insect (PDI); Waveform duration per event by insect (WDEi).
