# Supplementary material for: Risk assessment of ‘Candidatus Liberibacter solanacearum’ transmission by the psyllids Bactericera trigonica and B. tremblayi from Apiaceae crops to potato
Source: Sci Rep. 2017 Apr 3;7:45534. doi: 10.1038/srep45534 (PMC5377357; doi:10.1038/srep45534)
Supplement: Supplementary Information [file srep45534-s1.pdf]

Risk assessment of '*Candidatus* Liberibacter solanacearum' transmission by the psyllids *Bactericera trigonica* and *B. tremblayi* from Apiaceae crops to potato.

Authors

C. A. Antolinez, A. Fereres, A. Moreno*.

Supplementary information

Supplementary figures.

Figure S1. Scheme of the experimental design used in the two-choice assays for (A) *B. trigonica* and (B) *B. tremblayi*.

A.

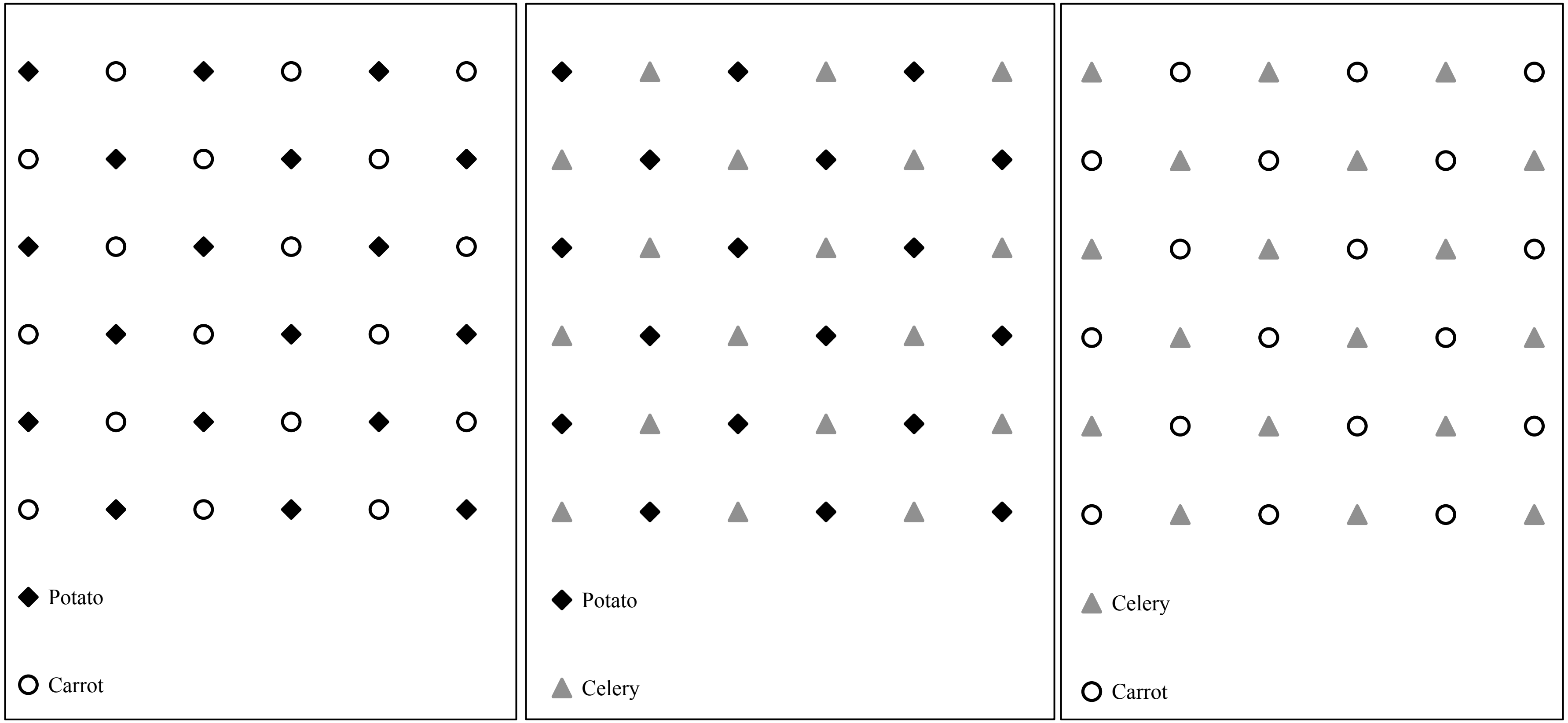

B.

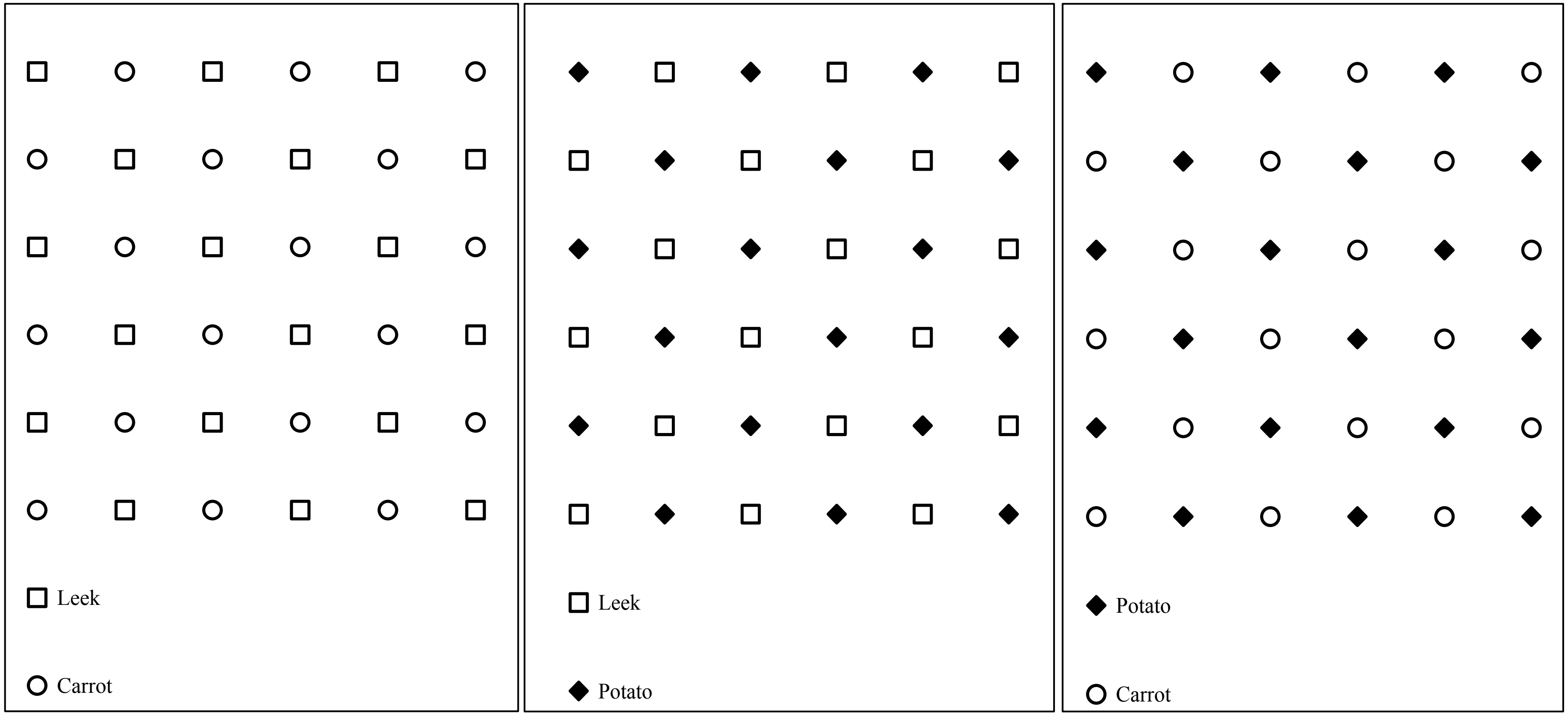

Supplementary Tables.

Table S1. Percentage of host plants infested by at least one psyllid of *B. trigonica* or *B. tremblayi* in non-choice assays. Different letters represent significant differences among treatments according to one-way ANOVA.

Non-choice	Potato	Carrot	Celery	P value
B. trigonica	25.00 ± 8.48 a	65.74 ± 7.91 b	49.07 ± 3.33 b	0.017
	Potato	Carrot	Leek	
B. tremblayi	21.29 ± 7.57 a	51.85 ± 3.33 b	57.40 ± 8.07 b	0.018

Table S2. Percentage of host plants infested by at least one psyllid of *B. trigonica* or *B. tremblayi* in dual-choice assays. Different letters represent significant differences among treatments according to Student`s t-tests.

Dual-choice	T1	P value	T2	P value	T3	P value
B. trigonica	Carrot = 72.22 ± 1.85 a	0.613	Celery = 66.66 ± 8.48 a	0.051	Potato = 24.07 ± 16.45 a	0.007
	Celery = 66.66 ± 8.48 a		Potato = 18.51 ± 5.55 b		Carrot = 74.07 ± 11.26 b	
	T1	P value	T2	P value	T3	P value
B. tremblayi	Carrot = 37.03 ± 4.89 a	0.550	Potato = 18.51 ± 4.89 a	0.007	Leek = 66.66 ± 0.00 a	0.250
	Leek = 59.25 ± 6.67 a		Carrot = 55.55 ± 4.55 b		Potato = 27.77 ± 11.11 b	
